# Phase Formation during Heating of Amorphous Nickel-Based BNi-3 for Joining of Dissimilar Cobalt-Based Superalloys

**DOI:** 10.3390/ma14164600

**Published:** 2021-08-16

**Authors:** Mojtaba Naalchian, Masoud Kasiri-Asgarani, Morteza Shamanian, Reza Bakhtiari, Hamid Reza Bakhsheshi-Rad, Filippo Berto, Oisik Das

**Affiliations:** 1 Advanced Materials Research Center, Department of Materials Engineering, Najafabad Branch, Islamic Azad University, Najafabad, Iran; Mojtaba.naalchian@gmail.com; 2 Department of Materials Engineering, Isfahan University of Technology, Isfahan 84156-83111, Iran; 3 Department of Materials Engineering, Faculty of Engineering, Razi University, Kermanshah, Iran; r.bakhtiari@razi.ac.ir; 4 Department of Mechanical and Industrial Engineering, Norwegian University of Science and Technology, 7491 Trondheim, Norway; 5 Structural and Fire Engineering Division, Department of Civil, Environmental and Natural Resources Engineering, Luleå University of Technology, 97187 Luleå, Sweden; oisik.das@ltu.se

**Keywords:** microstructural changes, mechanical properties, cobalt-based superalloy, transient liquid phase bonding

## Abstract

Phase transformations and the melting range of the interlayer BNi-3 were investigated by differential scanning calorimetry, which showed three stages of crystallization during heating. There were three exothermic peaks that indicated crystallization in the solid state. The cobalt-based X-45 and FSX-414 superalloys were bonded with interlayer BNi-3 at a constant holding time of 10 min with bonding temperatures of 1010, 1050, 1100, and 1150 °C using a vacuum diffusion brazing process. Examination of microstructural changes in the base metals with light microscopy and scanning electron microscopy coupled with X-ray spectroscopy based on the energy distribution showed that increasing temperature caused a solidification mode, such that the bonding centerline at 1010 °C/10 min included a γ-solid solution, Ni_3_B, Ni_6_Si_2_B, and Ni_3_Si. The athermally solidified zone of the transient liquid phase (TLP)-bonded sample at 1050 °C/10 min involved a γ-solid solution, Ni_3_B, CrB, Ni_6_Si_2_B, and Ni_3_Si. Finally, isothermal solidification was completed within 10 min at 1150 °C. The diffusion-affected zones on both sides had three distinct zones: a coarse block precipitation zone, a fine and needle-like mixed-precipitation zone, and a needle-like precipitation zone. By increasing the bonding temperature, the diffusion-affected zone became wider and led to dissolution.

## 1. Introduction

The nature of the crystallographic structure of nickel enables it to form a solid solution with many alloying elements and dissolve them. This mutual solubility has resulted in the creation of a large group of brazing alloys based on nickel. Nickel has a high melting point (1452 °C) and, consequently, the instability of metals at high temperatures inhibits the use of pure nickel as a brazing material. Adding melting point depressant elements, such as boron, silicon, carbon, and phosphorus, considerably reduces the brazing temperature of nickel. Moreover, various types of nickel-based filler metals are used in the bonding of different alloys depending on the type of application, due to nickel’s possibility of alloying with reinforcing elements such as chromium. Filler alloys are classified into Ni-Si-B, Ni-Cr-Si-B, Ni-P, and Ni-Cr-B systems based on the AWS A5.8 standard. The addition of a melting point depressant element and the reinforcement of alloying elements with other alloying elements, such as iron and molybdenum, are also undertaken in some cases [1]. Hot parts of gas turbines are usually repaired with nickel-based filler metals when heat and corrosion resistance are required [2]. It is possible to use these alloys in the forms of powder, paste, tape, wire, or foil. Currently, these alloys are fabricated by fast solidification processes (melt spinning), the main advantages of which are their flexibility and ease of use [3]. Also, they have other advantages, including uniformity in chemical and fuzzy compositions, higher penetration of the melting point reducing element, higher capillary activity, particularly narrow melting and freezing ranges, high bonding strength, excellent wettability, less dissolution of base metals during bonding, and much better melting properties than filler metals with a crystalline structure [4,5]. Ma et al. [6] employed the ultrasonic frequency induction method and micro-sized powders in crystalline and amorphous forms for the brazing of diamond powder to steel cylinders. They stated that amorphous nickel-based filler metals were heated and then formed crystalline grains with a mean grain size of 0.2–0.3 μm, while the mean grain size of the solid solution in the crystalline state was about 1 μm. They additionally claimed that the amorphous filler metal melted at a lower temperature and had a narrower melting range and, consequently, that diamond particles were uniformly distributed. Cylindrical tests revealed that brazed diamond particles with crystalline fillers were more easily removed from the amorphous state. Li et al. [7] compared TiAl-based brazing joints with amorphous and crystalline titanium-based filler metals. Examining the microstructure and performance of the joints enabled us to understand that amorphous filler metal has a more uniform microstructure, better melt distribution, and a narrower melting limit compared to the filler metal with a crystalline structure. Also, brazing joints with amorphous filler metal have always had higher shear strength than filler metal with a crystalline structure. The narrow melting range and lower melting point have led amorphous filler metals to be used in the form of foil, tape and even powder in transient liquid phase bonding. Hence, these metals reduce the amount of thermal damage to the base metals, including further crystallization, grain growth, precipitation dissolution, deposition of new phases, and local melting in the grain boundary. Brazing alloys have some limitations, including their limited stability in high-temperature applications, which causes the phenomenon of exothermic crystallization during heating. Amorphous alloys change their glassy state during heating and form stable phases, which make the thermal and crystallization properties of amorphous brazing foils highly suitable for carrying out brazing [8]. The crystallization temperature depends on the type and amount of metalloids (B, C, Si, and P) and the base metal. It should be noted that simultaneously adding silicon and boron enhances the crystallization temperature [9].

Amorphous Ni-based brazing alloys are extensively used in the brazing of cobalt- [10], nickel- [11], and iron -based [12] alloys. Also, examinations of the diffusion brazing of cobalt- [13], nickel- [14], and iron-based [15] alloys revealed that these processes are likely to achieve a bond strength near the strength of base metals. Cobalt-based superalloys are extensively applied in the power plant and gas turbine manufacture industries. They are used in the hot parts of gas turbines and can be badly damaged. Cobalt-based superalloys are fabricated and repaired using fusion welding, brazing, and transient liquid phase bonding processes. Rahmani et al. [16] used the GTAW process and L-605 filler metal to weld FSX-414 superalloys and examined the effect of welding on microstructure, tensile, and low-cycle-fatigue properties. They stated that the yield and ultimate stress of the welded samples were similar to those of a base metal, but there was low toughness in the welded samples. Also, the total fatigue life of the welded samples was much shorter than that of a base metal. Bakhtiari et al. [17] examined the transient liquid phase bonding of the FSX-414 superalloy with a BNi-9 filler metal at different temperatures and times. They argued that eutectic-like intermetallic compounds, such as nickel and chromium borides in the bonding zone centerline, significantly reduce the shear strength of the bond at low bonding temperatures and low holding times, and these destructive intermetallic compounds can be eliminated by increasing the temperature and time, leading the shear strength of the bonding to approach the strength of a base metal. Brazing or transient liquid phase bonding processes have been used to bond cobalt-based superalloys to nickel-based superalloys in order to increase the performance and longevity and produce a monolithic part or repair damaged parts. For example, Schoobaret et al. [18] examined the brazing of X-40 cobalt-based alloy and the IN738 nickel-based superalloy with a BNi-9 brazing alloy with a 50 μm gap joint. Studies conducted on their microstructures determined that there were chromium-rich borides, eutectic-like phases, and various carbides in the joint and that topologically compact phases were not formed during brazing in nearby base metals. They claimed that the high carbon percentage of the X-40 alloy was highly effective in inhibiting the formation of TCP phases during brazing and ensuing heat treatment. Abbasi-Khazaie et al. [19] investigated the transient liquid phase bonding of the FSX-414 cobalt-based superalloy to the IN738 nickel-based superalloy in order to improve the function of first-stage nozzles and rotating turbine blades. They argued that selecting optimal bonding parameters is highly effective in eliminating destructive intermetallic compounds of the central bonding line, preventing local melting in the base metals, and achieving a single-phase zone in the bonding region.

No studies have been conducted on the dissimilar bonding of cobalt-based superalloys. This study was conducted in order to examine the microstructural changes and phase transformation during heating of the BNi-3 amorphous interlayer to the bonding temperature in order to understand the solidifying sequence and its influence on dissimilar brazing of X-45 and FSX-414 cobalt-based alloys. Therefore, the behavior of a Ni-Si-Fe-B-based amorphous alloy generally and as a bonding factor was studied with differential scanning calorimetry.

## 2. Materials and Methods

In this study, the cobalt-based ingots X-45 and FSX-414 were used as base metals in as-cast conditions and their chemical compositions were analyzed with an optical spectrometer, which was calibrated based on that the chemical compositions given in Table 1. An amorphous BNi-3 interlayer nickel-based foil with a thickness of 50 μm was used as filler metal to diffusively bond the base metals; the chemical composition is given in Table 2. The wire electric discharge machine-cut the samples with dimensions of 10 × 10 × 5 mm^3^ from the X-45 and FSX-414 casting ingots, and all the surfaces were ground with SiC sandpaper with numbers 600–1200, cleaned in an ultrasonic acetone bath for one hour, and kept in alcohol until bonded. The amorphous BNi-3 interlayer with dimensions of 10.5 × 10.5 mm^2^ was cut, cleaned in an ultrasonic acetone bath, and kept in alcohol. The transient liquid phase bonding process of base metal was undertaken with a full overlap design. Therefore, the filler metal was placed between the base metals, and a suitable fixture with a low pressure was used to hold them to prevent pieces from moving and the overflow of the interlayer melt during the bonding process. A heat-resistant chromium–molybdenum-based steel fixture was used in order to be able to withstand high temperatures. Diffusion bonding was conducted in a vacuum furnace with a vacuum degree of 2 × 10^−6^ Torr. The heating rate was 15 °C/min. Bonding temperatures of 1010, 1050, 1100, and 1150 °C and a holding time of 10 min were used for diffusion bonding of the transient liquid phase of base metals. At the end of the bonding operation, samples with dimensions of 10 × 10 × 10 mm^3^, prepared for the characterization operation, were obtained.

The TLP-bonded samples were cut perpendicular to the joint gap, and metallography was undertaken according to the ASTM E3 standard. The mounted samples were ground with 60–3000 grit sandpaper and were initially polished with 1 μm alumina particles, then finally polished with 0.05 μm alumina particles. In the microstructure investigation of the base metals in the cast and bond states, Murakami solution with $10\;g\;\mathrm{KOH}+10\;g\;K_{3}{\mathrm{Fe}(\mathrm{CN})}_{6}+100\;{\mathrm{mL}\;H}_{2}O$ was used, and, to investigate the microstructure of the bonded zone, waterless Kalling’s solution with 100 mL HCl + 100 mL ethanol + 4.5 g CuCl_2_ was used. An Axiotech Zeiss optical microscope (ZEISS, Munich, Germany), equipped with Image J image analysis (National Institutes of Health, Bethesda, MD, U.S.A.), was used to image and determine the bonding region and the measurements of the bonded zone. An FEI Accura TM850 + field emission scanning electron microscope (FESEM, Thermo Fisher Scientific, Waltham, MA, U.S.), equipped with X-ray spectroscopy based on the energy distribution of the Oxford Instruments INCA 400 X-Max model (Asylum Research, Abingdon, U.K.) was used to image and determine the chemical composition of the phases in different regions of the bonded samples. X-ray diffraction patterns of base metals in casting and bonded states were obtained using a PHILIPS PW3040 (Rigaku Americas Corporation, The Woodlands, TX, U.S.) at a wavelength of 1.54 Å. The melting temperature and metallurgical phenomena were recorded using differential scanning calorimetry (449F Netzsch, NETZSCH, Selb, Germany). Samples with diameters of 5 mm were obtained by punching from the interlayer and placed in the alumina crucible of the machine. A heating rate of 15 °C/min and a cooling rate of 10 °C/min under pure argon flow (50 mL/min) were used [20].

## 3. Results and Discussion

### 3.1. Phase Transformation in Nickel-Based Amorphous BNi-3 Interlayer

**Figure 1:**
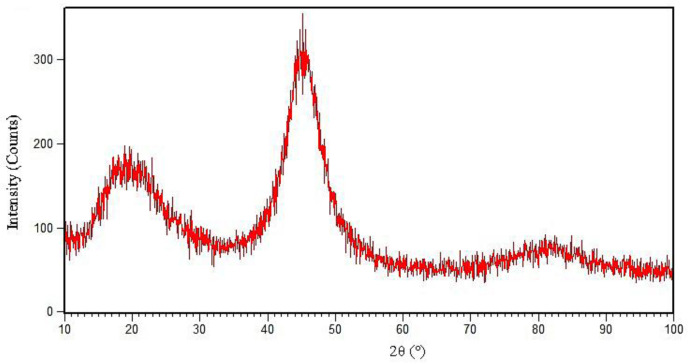
XRD pattern of BNi-9 amorphous interlayer foil with composition Ni_78.92_Si_7.54_B_13.07_Fe_0.45_.

**Figure 2:**
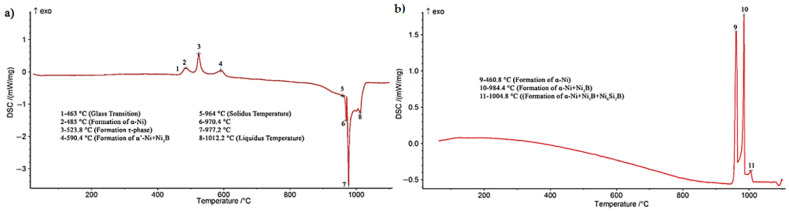
DSC trace during (**a**) heating and (**b**) cooling from 1100 °C for BNi-3 foil alone, showing transformation phenomena at each stage.

In this research, an amorphous BNi-3 interlayer with a chemical composition of Ni-4.23Si-2.82B-0.51Fe was used, which is equivalent to Ni-13.07B-7.54Si-0.45Fe (at. %), which can be represented as Ni_78.92_Si_7.54_B_13.07_Fe_0.45_. Figure 1 presents the X-ray diffraction pattern of the amorphous BNi-3 interlayer. The diffraction pattern obviously shows that the interlayer was in an amorphous state. The wide peaks indicate an amorphous state. Dong et al. [21] applied an amorphous Ti_60_Ni_22_Cu_10_Zr_8_ interlayer in the brazing of TiAl alloy to Cr40 steel. They declared that the lack of sharp peaks in positions corresponding to the crystal structure in the X-ray diffraction pattern indicate an amorphous property that is effective in improving the wettability, diffusion, and optimal bonding properties. The melting and solidification behavior of the interlayer in the amorphous and crystalline states, the microstructural revolution and melting range, the activation energy, and the metallurgical phenomena were examined using a scanning differential calorimetry test. Figure 2 indicates the path of the differential scanning calorimetry during heating and cooling of the interlayer. As Figure 2a shows, first there was an interlayer in an amorphous and glassy state. Glass metals were converted into crystalline amorphous form by passing the glass transition temperature (Tg), which occurred at 436 °C. There were three exothermic peaks that indicate crystallization in the solid state. The amorphous alloys were crystallized by nucleation and growth, which heavily depend on the heating rate [22]. These three exothermic peaks represent three stages with different activation energies during crystallization in conditions away from the equilibrium state. These three exothermic peaks occurred at temperatures of 485, 523.8, and 590.4 °C, which was in agreement with the formation of new phases in the interlayer. The conditions of each stage were simulated in scanning differential calorimetry and X-ray diffraction analysis in order to examine the various stages of crystallization and the types of phases.

#### 3.1.1. The First Stage of Crystallization: Formation of Solid-Solution Crystals Based on Nickel (α-Ni)

**Figure 3:**
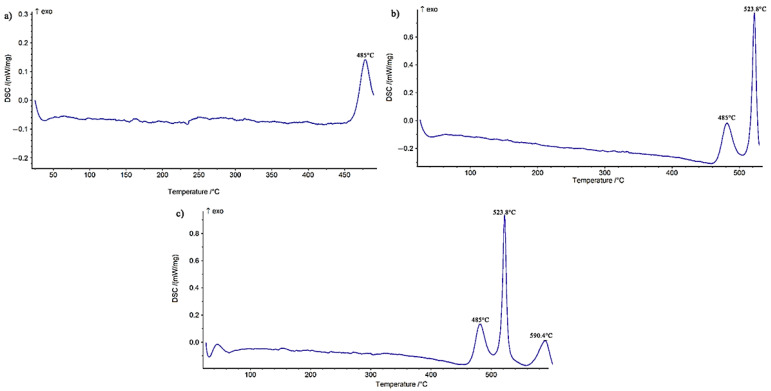
DSC trace of BNi-3 amorphous foil after annealing: (**a**) 490 °C/10 min, (**b**) 530 °C/10 min, and (**c**) 590 °C/10 min.

**Figure 4:**
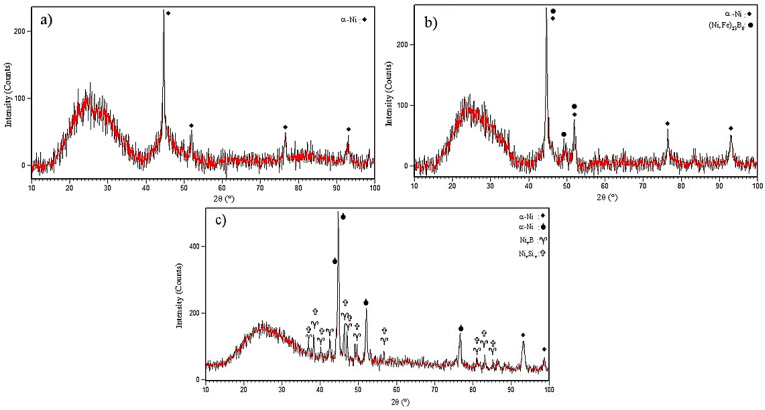
XRD phase analysis of BNi-3 interlayer after annealing under the following conditions: (**a**) 490 °C/10 min, (**b**) 530 °C/10 min, and (**c**) 590 °C/10 min.

As Figure 2a shows, the first peak was formed in the temperature range of 463–508 °C, with a maximum temperature of 485 °C. Accordingly, the interlayer was heated to 490 °C and stored for 10 min in a differential scanning calorimeter to complete the first-stage transformation. Figure 3 presents the differential scanning calorimetry path of the annealed foil at 490 °C/10 min, 530 °C/10 min, and 590 °C/10 min. The heat energy released in the first step was measured as 13.21 j/g (Figure 3a). Figure 4 shows XRD phase analysis of BNi-3 interlayer after annealing under different conditions 490 °C/10 min, 530 °C/10 min, and 590 °C/10 min. Figure 4a shows the X-ray diffraction pattern of the annealed sample at 490 °C/10 min. Analysis of the X-ray diffraction pattern revealed that there was an α-Ni phase (nickel-rich solid solution) and it was determined, in light of the intensity and number of diffraction peaks, that a significant part of the amorphous alloy had been transformed into the crystalline phase. A wide peak at the beginning of the X-ray diffraction pattern was caused by either the presence of an amorphous state or a very small (nanometer) particle size in the crystal structure. The α-Ni solid-solution lattice parameter of 0.3534 ± 0.05 nm was calculated based on the Nelson–Riely method. The pure nickel lattice parameter, which was caused by the presence of elements such as iron, silicon, carbon, and boron, was 0.35157 nm. Passing the glass transition temperature results in the formation of extremely small nickel crystals in the amorphous matrix, which causes the epitaxial growth of nickel crystals on the previously formed nucleation when the temperature range of the crystallization stage is reached [23]. The crystallization process of the first stage, governed by the influence of the solid state at low temperatures, requires much time; accordingly, the wide peak in the X-ray diffraction pattern indicated that the amorphous state had not been thoroughly transformed into a crystalline structure and required more time.

#### 3.1.2. The Second Stage of Crystallization: Formation of a Metastable τ-Phase with M_23_B_6_-Type Structure

As Figure 2a shows, the second stage of the crystallization process of the interlayer is in the temperature range of 508–554.3 °C, with a maximum temperature of 523.8 °C. Accordingly, the interlayer was heated to 530 °C for 10 min in a scanning differential calorimeter to complete the second stage of crystallization. Figure 3b depicts the differential scanning calorimetry path of the foil annealed at 530 °C/10 min. The heat energy released in the second step was measured as 15.34 j/g. Figure 4b displays the X-ray diffraction pattern of the annealed interlayer at 530 °C/10 min. It is obvious that there was a new phase τ, according to the M_23_B_6_ (M = Ni, Fe) stoichiometric formula, along with the α-Ni phase (nickel-based solid solution). Kurakova et al. [24] conducted a study on the crystallization process of nickel-based amorphous alloys and found that a solid solution of nickel-based alloys was formed and a metastable phase τ was then created, which disintegrated into other components after passing this stage. The α-Ni phase that was formed included relatively significant amounts of silicon (the solubility of silicon in nickel is 12 at. % [25]) and had no boron (the solubility of boron in nickel is approximately zero in the temperature range from 400 to 600 °C [26]). Consequently, the rest went to the grain boundaries and reacted with nickel and iron to form the metastable phase τ.

#### 3.1.3. The Third Stage of Crystallization: Formation of Ni_3_B and Ni_3_Si

As Figure 2a shows, the third step of the crystallization process of the interlayer was in the temperature range of 554.3–620 °C, with a maximum temperature of 590.4 °C. Accordingly, the interlayer was heated to 590 °C for 10 min in a scanning differential calorimeter to complete the third stage of crystallization. Figure 3c shows the differential scanning calorimetry path of the annealed sample at 590 °C/10 min. The thermal energy released in the third step was measured as 11.76 j/gr. Figure 4c shows the X-ray diffraction pattern of the interlayer annealed at 590 °C/10 min. Two types of nickel-based phases were identified in this diffraction pattern: (a) a nickel-based solid solution (α-Ni) that was formed in the first stage and (b) a nickel-based solid solution (α′-Ni) that was caused by metastable phase decomposition of τ. The lattice parameter of α′-Ni was calculated to be 0.3527 ± 0.05 nm. The presence of Ni_3_B and Ni_3_Si_2_ peaks indicated that the τ phase had been completely transformed, so that its peaks totally disappeared in the X-ray diffraction pattern. In their study, Inoue et al. [27] found that the metastable phase τ was transformed in the third stage through the eutectoid reaction τ→α′-Ni + Ni_3_B and the silicon element was rejected to the α-Ni phase by the Ni_3_B phase and precipitated by the solid-state transformation of the Ni_3_Si_2_/Ni_3_Si.

**Figure 5:**
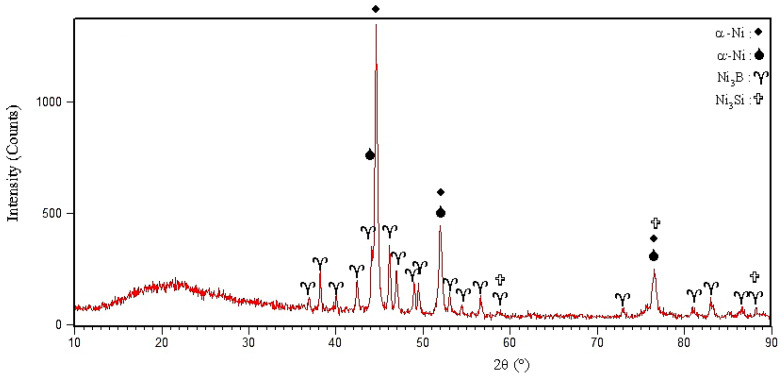
XRD phase analysis of the BNi-3 interlayer after annealing at 620 °C/10 min.

**Figure 6:**
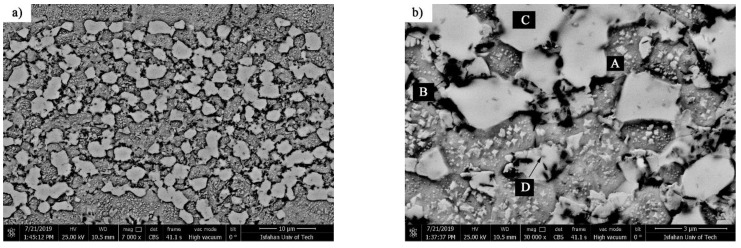
The scanning electron microscopy (SEM) microstructure of the interlayer annealed at 980 °C for 10 min: (**a**) low and (**b**) high magnification. Note: (A) α-Ni, (B) α′-Ni, (C) Ni_3_B and (D) Ni-Si-B compounds.

The crystallization process terminates at 620 °C; consequently, the interlayer was annealed at 620 °C/10 min to identify the phases formed at the end of the crystallization process. As Figure 5 shows, the α-Ni, α′-Ni, Ni_3_B, and Ni_3_Si were identified in the X-ray diffraction pattern, indicating the completion of the crystallization process. The temperatures of the solidus and liquidus of the interlayer are 964 and 1012.3 °C, respectively. Accordingly, the interlayer was heated at 980 °C for 10 min in order to examine the phases created in solid-molten conditions. Figure 6a shows the scanning electron micrograph of the interlayer heated to 980 °C for 10 min. It is possible to observe various precipitations with different morphologies, which Figure 6b shows with more magnification. Table 3 presents the EDX analysis of the phases shown in Figure 6b. The gray zone A included significant amounts of the nickel element, which indicated a nickel-rich solid solution (α-Ni). This solidus solution included silicon, so the solubility of silicon in nickel was 12 at. %. The dark zone B included significant amounts of nickel with very low amounts of silicon (6 at. %). This phase was a nickel-rich solid solution (α′-Ni) that was precipitated from the eutectoid transformation τ→α′-Ni + Ni_3_B. The white zone C comprised a significant part of the surface of the transformed interlayer and was located higher than the dark area of the background. This phase was rich in nickel and boron, which included very low amounts of silicon. Consequently, it is possible to state that the white phase was Ni_3_B, which is nickel-rich boride. Also, the silicon-rich precipitations (25 at. %) containing boron in nickel-rich boride zones (zone D in Table 3) were identified and seemed to be Ni-Si-B compounds.

It is possible to express the solidification path of the interlayer with a nickel–silicon–boron phase diagram [28] based on the differential scanning calorimetry curve for the cooling of the interlayer melt: the melt of the interlayer began to solidify at a temperature of 1004.8 °C and α-Ni phase dendrites were formed. This nickel-rich phase moved the chemical compound of the melt towards the nickel–boron binary eutectic by rejecting the boron element to the remaining melt that was formed through the eutectic reaction at 984.4 °C in the α-Ni + Ni_3_B phases. Ultimately, the enrichment of the remaining melt with boron and silicon formed α-Ni + Ni_3_B + Ni_6_Si_2_B phases through a ternary eutectic reaction at 960.8 °C.

### 3.2. Microstructure of Brazed Joints

**Figure 7:**
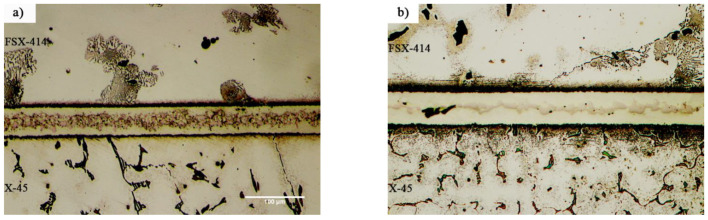

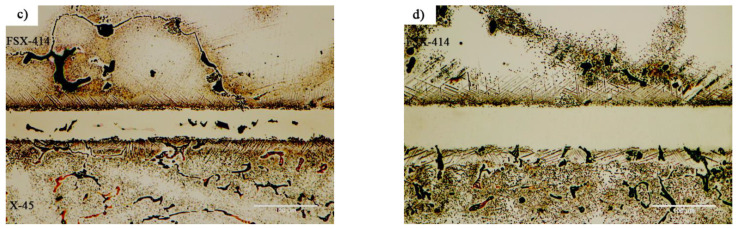
Optical microscopy of microstructural evolution of vacuum brazed at constant holding times of 10 min at different temperatures: (**a**) 1010 °C, (**b**) 1050 °C, (**c**) 1100 °C, and (**d**) 1150 °C.

**Figure 8:**
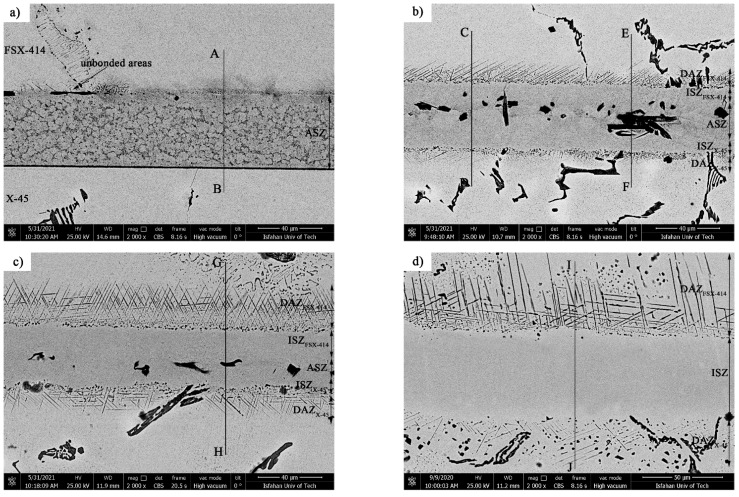
The SEM microstructure of the TLP-bonded specimen at a constant holding time 10 min and different temperatures: (**a**) 1010 °C (A–B Line Scan), (**b**) 1050 °C (C–D and E–F Line Scan), (**c**) 1100 °C (G–H Line Scan), and (**d**) 1150 °C (I–J Line Scan).

**Figure 9:**
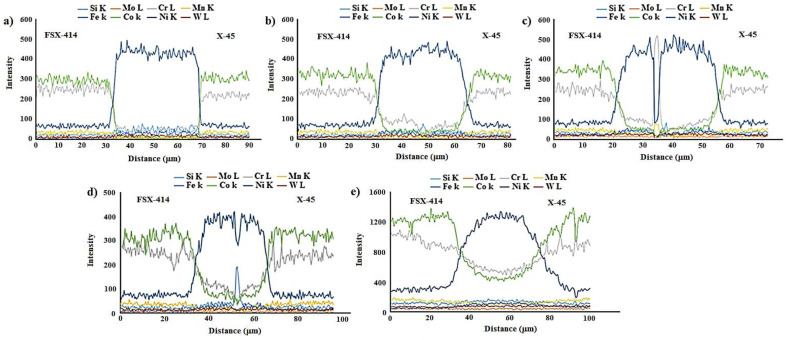
Line scans across the bonded zone at a constant time of 10 min and different temperatures: (**a**) 1010 °C, (**b**,**c**) 1050 °C, (**d**) 1100 °C, and (**e**) 1150 °C.

Figure 7 and Figure 8 show images from obtained with optical and scanning electron microscopy of the microstructure of the brazed joints at temperatures of 1010, 1050, 1100, and 1150 °C with a constant holding time of 10 min. In fact, this image explains the effect of temperature on the bonding of the transient liquid phase of the X-45 and FSX-414 cobalt-based superalloys with the BNi-3 interlayer. As Figure 7a shows, although bonding took place at a temperature lower than the liquidus temperature of the interlayer, the two base metals were bonded together and there was a misbonded zone. Figure 8a presents an SEM micrograph for the microstructure of the bonded area at 1010 °C/10 min. It is obvious that 93% of the bonding zone included globular-like intermetallic compounds. There were dense regions in the proximity of the bonding zone in the base metals, indicating solid-state diffusion before the liquidus temperature of the interlayer was reached. Figure 9a presents the linear analysis for the bonded zone of the sample at 1010 °C/10 min (A–B). There was a constant concentration of nickel in the base metals X-45 and FSX-414 which would increase suddenly when the interface of the base metals/bond region was reached due to the ineffectual vacillations in the bond zone. There was an essentially constant concentration of cobalt in the base metals with very few changes. The maximum concentration of cobalt was in the interface of the base metals with the bonding zone and the lowest concentration was in the bounding zone. There was a highly interesting point in the linear analysis of the sample at 1010 °C/10 min regarding chromium. The BNi-3 interlayer included only boron and silicon as melting point depressant elements. The bonding temperature was lower than the liquidus temperature of the interlayer at this condition, and there was not enough time for the solid-state diffusion and dissolution of the base metals in the small melt of the interlayer. Hence, the lowest chromium concentration was in the bonding zone, and alloying elements aggregated as precipitation increased their local concentration.

Figure 7b shows images obtained with optical microscopy of the microstructures of the bonded samples treated at 1050 °C for 10 min. The microstructures were different from the samples treated at 1010 °C/10 min. An increase 40 °C caused the microstructure of the central bonding line to be changed from a globular-like state to a continuous and long shape. Figure 8b shows an SEM micrograph of the bonded zone of the sample at 1050 °C/10 min. Precipitations, including constant and distinct phase contrast, formed new phases. Linear analysis was performed for the bonding seam, including two phases (C–D and E–F), as shown in Figure 9b,c. Figure 9b shows that the nickel concentration reached its maximum at a short distance from the interface of the base metal/bonded zone in the bonding centerline. This issue was also noted in the chromium element (Figure 9c). There was an interesting issue regarding the entry of chromium from base metals into the interlayer melt and its collection in the form of a chromium-rich phase in the bonding central line. Figure 7c shows a light microscopy image of the microstructure of the bonded sample treated at 1100 °C/10 min. It was obvious that there were some intermetallic compounds from the remaining melt of the interlayer, but the precipitations came out of the constant state and could be observed discontinuously in the centerline of the bounding as narrow bands. Linear analysis for the joint showed that the distance between the concentrations of nickel and chromium, from the interface to the central line of the joint, increased with slight but constant fluctuations. Also, the concentration of cobalt increased significantly, which indicated the diffusion and dissolution of base metals in the melt of the interlayer (G–H, Figure 9d). Figure 7d presents images obtained with optical microscopy of the microstructure of the bonded sample treated at 1150 °C/10 min. The bonding zone had no eutectic-like compounds and indicated that a single-phase zone had been achieved. Linear analysis for the joint (I–J, Figure 9e) indicated that the concentrations of cobalt, chromium, tungsten, and molybdenum in the bonding zone had increased and these elements had been replaced as substitutes in the nickel crystal lattice. On the other hand, alloy elements were more uniformly distributed. The formation of a single-phase zone by keeping the interlayer melt at a constant temperature explains the isothermal solidification of the interlayer melt (Figure 8d).

It is possible to state, based on microstructural observations and linear analysis, that the concentration gradient along the joint seam caused a microstructural gradient. Consequently, according to Figure 8, it is possible to divide the bonded zone into seven microstructural zones, which we list from top to bottom: (1) FSX-414 cobalt-based superalloy, (2) diffusion-affected zone on the FSX-414 side (DAZ_FSX-414_), (3) isothermal solidification zone on the side of FSX-414 (ISZ_FSX-414_), (4) athermal solidification zone (ASZ), (5) isothermal solidification zone on the side of the superalloy X-45 (ISZ_X-45_), (6) diffusion-affected zone on the X-45 side (DAZ_X-45_), and (7) X-45 cobalt-based superalloy.

**Figure 10:**
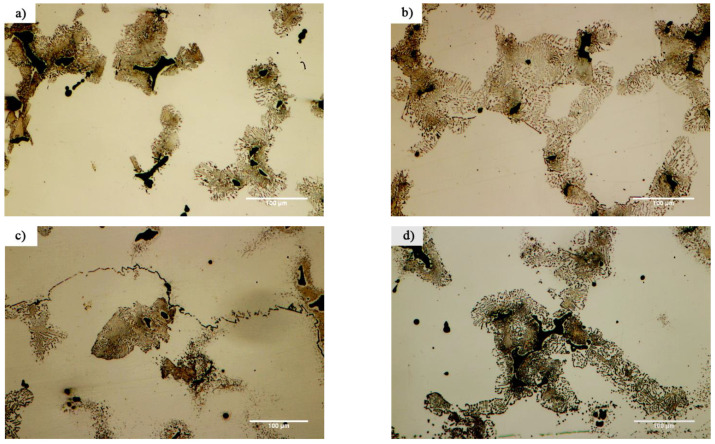
Optical microscopy of microstructural evolution of FSX-414 at a constant time of 10 min and different temperatures: (**a**) 1010 °C, (**b**) 1050 °C, (**c**) 1100 °C, and (**d**) 1150 °C.

**Figure 11:**
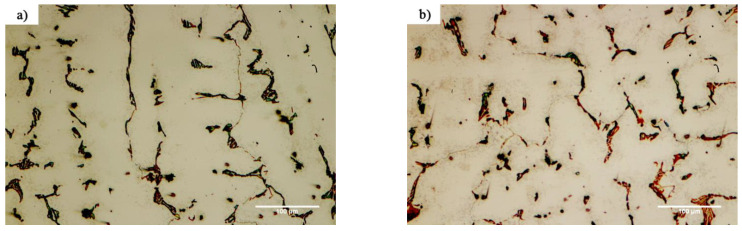

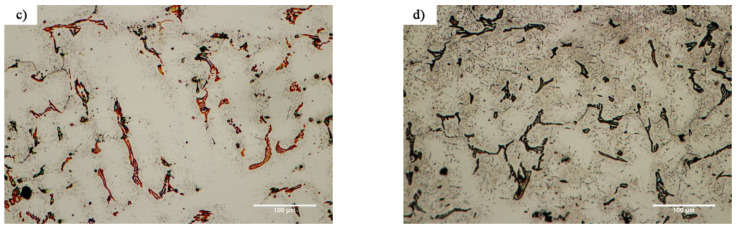
Optical microscopy of microstructural evolution of X-45 at a constant time of 10 min and different temperatures: (**a**) 1010 °C, (**b**) 1050 °C, (**c**) 1100 °C, and (**d**) 1150 °C.

**Figure 12:**
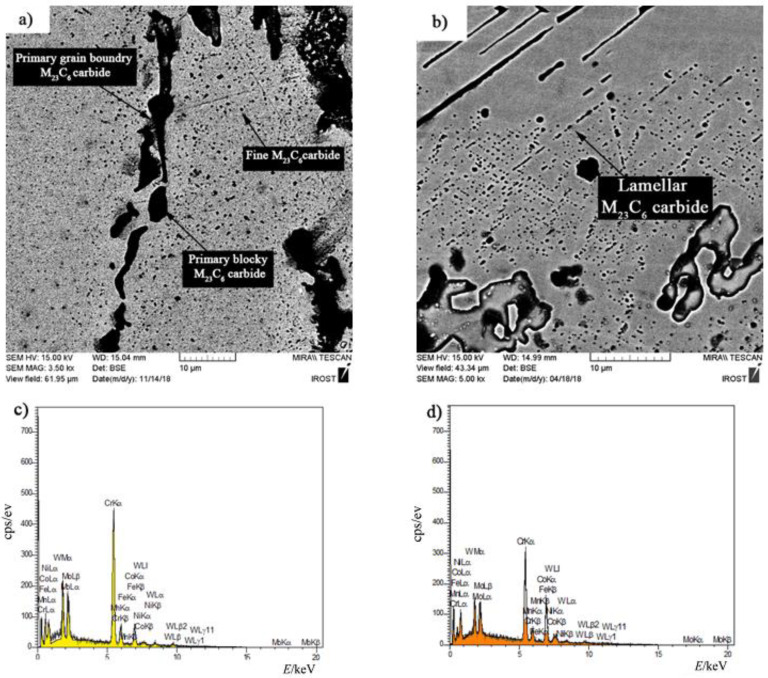
SEM micrographs of (**a**) fine M_23_C_6_ carbide and (**b**) lamellar M_23_C_6_ carbide and (**c**,**d**) EDX spectra of the corresponding phases.

Figure 10 shows the microstructural evolution in the FSX-414 superalloy at a fixed time of 10 min and temperatures of 1010, 1050, 1100, and 1150 °C. Carbides with eutectic morphology can maintain their stability, and there were no traces of secondary phase particles in the rich field of cobalt (α-Co), indicating that increasing the amount of chromium by 4 wt.% could ensure the temperature stability of the FSX-414 superalloy was maintained. Wei et al. [29] examined the microstructural changes of a 6509 K superalloy at different temperatures with various holding times. They argued that cobalt-based cast alloys are thermodynamically unstable and that their stability completely depends on the temperature. At high temperatures, M_23_C_6_ carbides comprise the most stable carbide state in the conditions of service. Accordingly, if carbides are deposited as eutectic islands, their temperature stability will be increased [30]. There were completely different microstructural conditions in the X-45 cobalt-based superalloy. Figure 11 shows the microstructural changes in the X-45 superalloy at a fixed time of 10 min and temperatures of 1010, 1050, 1100, and 1150 °C. It is obvious that increasing temperature caused the dark particles to be deposited in the matrix. These precipitations were deposited as the kind of clouds that have a maximum volume fraction around the grain boundaries and interdendritic zones, and their amount decreased in the center of cobalt-rich grains (α-Co). Figure 12 exhibited SEM micrographs and EDX spectra of X-45 superalloy. Figure 12a shows an SEM micrograph of fine particle deposition around the initial X-45 superalloy carbides at 1150 °C/10 min. EDX analysis of the deposits (Figure 12c) indicated that they were rich in chromium. Examining the chemical analysis and previously published papers [31,32], we judged that these particles were M_23_C_6_ carbides. In fact, these were secondary M_23_C_6 (_M = Co, Cr, W, Mo) carbide particles that had been deposited during the cooling of the room temperature due to solid-state diffusion. This type of carbide particle is precipitated in aging conditions (deposition in the standard heat treatment conditions of 980 °C/240 min) or during high-temperature service in cobalt-based superalloys [30], nickel-based superalloys [33], and even steels [34]. These precipitations are called secondary carbides and are formed in situ and have a particle, cube, or disc morphology. Also, it was possible to observe a series of deposits with a dotted morphology at 1150 °C/10 min in the X-45 superalloy at a distance of 50 μm from the interface of the bonding zone, as shown in Figure 12b. EDX analysis showed a chemical composition similar to deposits (Figure 12d). This morphology of M_23_C_6_ is named lamellar carbide and it has been observed in the DZ40M alloy [35] and in a Co-Cr-Mo-C base alloy [36]. In their study, Gui et al. [35] stated that the released atoms were absorbed during heating by stacking faults in the cobalt matrix and local segregation zones were created. M_23_C_6_ carbides nucleate and grow a lamellar character due to the in situ reaction between chromium and carbon in local segregation situations.

**Figure 13:**
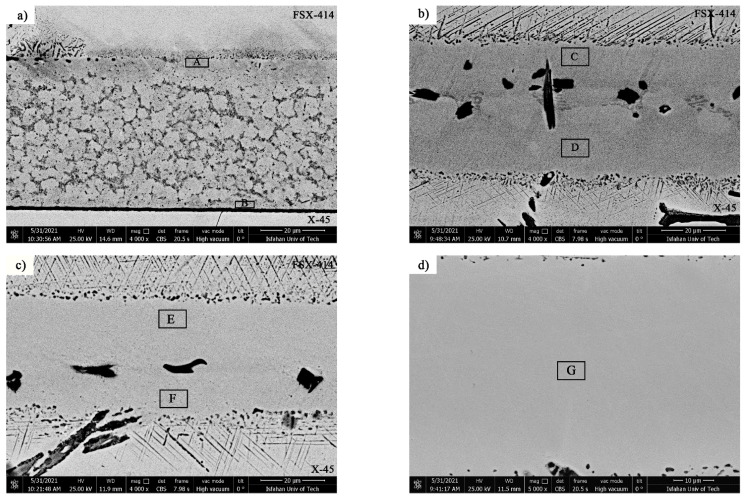
SEM microstructural revolutions of the bonding zones at a constant holding time of 10 min and different temperatures: (**a**) 1010 °C (A = ISZ_FSX-414_ and B = ISZ_X-45_), (**b**) 1050 °C (C = ISZ_FSX-414_ and D = ISZ_X-45_, (**c**) 1100 °C (E = ISZ_FSX-414_ and F = ISZ_X-45)_, and (**d**) 1150 °C (G = ISZ).

Figure 13 shows SEM micrographs of TLP-bonded samples with greater magnification at temperatures of 1010, 1050, 1100, and 1150 °C and a constant holding time of 10 min. The dense microstructure with a globular-shaped appearance for the 1010 °C/10 min treatment (Figure 13a) indicated the simultaneous existence of melt and solid together, and the conditions for the complete melting of the crystallized components in the interlayer were not present, as the liquidus temperature of the interlayer was 1012.2 °C. The amount of the formed molt that, among the particles’ crystallized phases, reached the middle layer/base metal interfaces through capillary force was very small, and, through only a little diffusion of the melting point depressant elements into the base metals, very narrow regions of the isothermal solidification zone were created in close proximity to the interfaces, and the rest of the molten components and crystallized components were athermally solidified. Regarding the nickel–silicon–boron phase diagram, the first phase, composed of a solid/liquid joint interface as epitaxial growth on the base metal grains, was a nickel-rich γ proeutectic solid solution formed as a narrow band on both sides of the interface of the base metals inside the bonding zone. Chemical analysis of the EDX data for ISZ_FSX-414_ (A in Table 4) and ISZ_X-45_ (B in Table 4) indicated the formation of a nickel-rich solid solution. The existence of very small amounts of the alloying elements chromium, tungsten, and molybdenum indicated the absence of sufficient molt for the dissolution of base metals. In the second step, a binary eutectic of Ni-rich boride and Ni-rich c solid solution was formed. The growth of nickel-rich dendrites from the interface of the molten interlayer/base metals enriched the residual molt with elements (boron and silicon) with a distribution coefficient smaller than the unit. The low solubility of boron in nickel (0.3 at. % based on a binary nickel–boron phase diagram [37]) and the low boron distribution coefficient in nickel (0.008 based on a binary nickel–boron phase diagram [37]) caused the rejection of the boron element to adjacent areas of nickel-rich dendrites.

**Figure 14:**
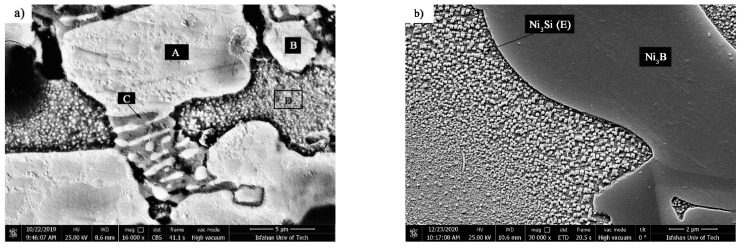
SEM microstructures of the TLP-bonded specimens with the 1010 °C/10 min condition: (**a**) athermal solidification zone: (A) Ni_3_B, (B) Ni_3_B, (C) Ni_6_Si_2_B, (D) α-Ni and (**b**) solid-state precipitation of Ni_3_Si.

**Figure 15:**
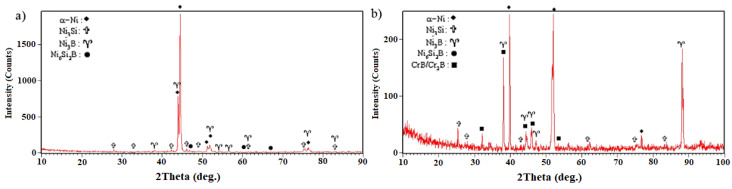
XRD patterns of fracture surfaces with different conditions: (**a**) 1010 °C/10 min and (**b**) 1050 °C/10 min.

This unequal solute rejection caused the residual molten chemical composition to move towards the eutectic chemical composition of nickel–boron and then followed the eutectic line of γ and Ni_3_B. Figure 14 shows the SEM micrograph of the athermal solidification zone of the 1010 °C/10 min sample with a higher magnification. Point EDX analysis from the island phase with an irregular shape (A in Table 5) and the distributed fine phase (B in Table 5) revealed that these phases were rich in nickel and the presence of the boron element was confirmed. Thus, accumulation of the nickel and boron elements together indicated a nickel-rich phase, which was likely to be nickel-rich boride (Ni_3_B). In the third step, ternary eutectic nickel–boron–silicon was formed. Due to the lack of solubility of silicon in nickel-rich boride, the formation of the boride phase was associated with the rejection of the silicon element in the residual molten. A distribution coefficient smaller than the unit in nickel enriched the residual molt with a silicon element. Although boron was consumed in the previous steps, there was still some of it in the final molt. Hence, simultaneous enrichment of the chemical compound with boron and silicon caused the chemical composition to move towards the ternary eutectic and the formation of γ + Ni_3_B + Ni_6_Si_2_B. Based on Figure 14a, this phase was formed of the residual molten phase in the last step; hence, it precipitated among the Ni_3_B phases. Point analysis of this phase showed that it included significant amounts of nickel and silicon and, in addition, a boron element was detected (C in Table 5). Thus, it can be claimed that it was probably Ni_6_Si_2_B. EDX area analysis (D in Table 5) indicated that it included a considerable amount of silicon (4.24 wt.%). Figure 14b shows an SEM micrograph of the microstructure of the nickel-rich matrix at a greater magnification. In the nickel-rich matrix, particles that had cubic shapes and a size of 200 nm were regularly distributed. Chemical analysis of the EDX data showed that they were rich in nickel and silicon (E in Table 5). The precipitation method and particle size indicated that these precipitations were deposited during cooling due to solid-state diffusion. The silicon value of these particles was about 12.2 at. %. Considering the nickel–silicon binary phase diagram [37], the solubility of silicon in nickel at 1050 °C is about 15 at. %, while its solubility at room temperature is 8 at. %; therefore, while cooling from bonding temperature to room temperature, this difference in solubility caused the precipitation of fine particles, which were probably Ni_3_Si. The X-ray diffraction pattern from the fracture surface of the 1010 °C/10 min and 1050 °C/10 min sample is shown in Figure 15. It is strongly evident that the γ, Ni_3_B, Ni_6_Si_2_B, and Ni_3_Si phases were formed in the molten and solidified interlayer (Figure 15a). It should be noted that, in the X-ray diffraction pattern, the chromium-rich boride phase was not detected, which might have been related to there being a very low amount of it or it not forming at all.

**Figure 16:**
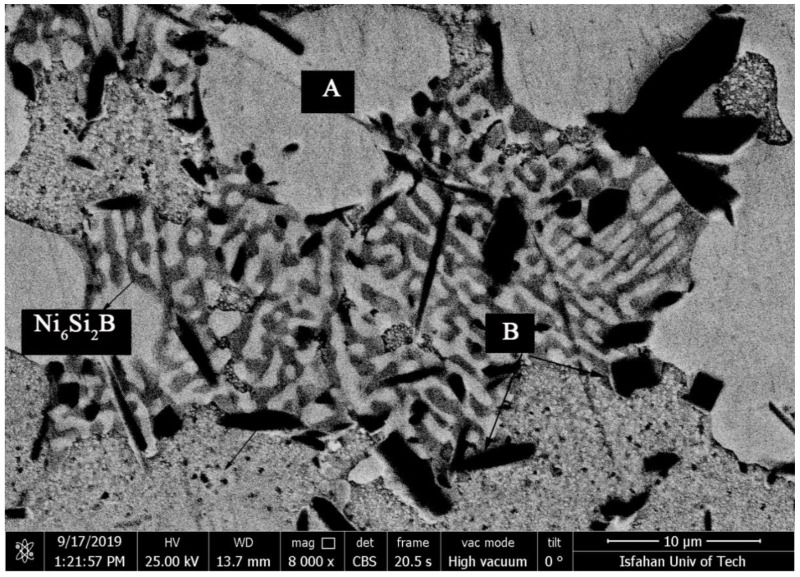
SEM image of the athermal solidification zone microstructure of the TLP-bonded specimen with the 1050 °C/10 min condition. Note: (A) Ni_3_B and (B) CrB/Cr_2_B.

Figure 13b shows the SEM micrograph of the TLP-bonded sample at 1050 °C/10 min. The extent of the isothermal solidification zones on both sides of the interface considerably increased. EDX analysis of ISZ_FSX-414_ and ISZ_X-45_ (C and D, respectively) showed the entry of chromium, cobalt, tungsten, and molybdenum elements from the base metals to the bonding area (Table 4). Increasing the extent of the nickel-rich isothermal solidification zone through increasing temperature revealed an increase in the diffusion rate of the melting depressant elements (boron and silicon) to base metals. Although the extent of the athermal solidification zone was reduced, its microstructure was generally altered. New precipitations were formed with discontinuous morphologies and continuous morphologies. Figure 16 shows the SEM micrograph of the athermal solidification zone of the 1050 °C/10 min sample with greater magnification. EDX analysis of phase A (Table 6) showed that it was rich in nickel. EDX analysis of phase B (Table 6) showed that it was rich in chromium. Thus, the formation of chromium-rich boride was confirmed. Regarding microstructural observations, the solidification mechanism in the 1050 °C/10 min sample was completely different from that of the 1050 °C/10 min sample. The nickel, chromium, silicon, and boron changed the solidification mode. As the ternary phase diagrams of nickel–chromium–boron [38] and nickel–silicon–boron [28] show, the first phase that grew dendritically from the solid/molten interlayer interface was the nickel-rich solid-solution proeutectic phase. The maximum solubilities of boron, chromium, and silicon in nickel are 0.3, 15, and 47 at. % (at eutectic Ni-Cr temperature), respectively; consequently, the unsteady-state rejection of these elements enriched the remaining melt of chromium, boron, and silicon. As the binary nickel–boron and chromium–boron phase diagrams [37] show, the binary eutectic temperatures of the formation of the nickel-rich and chromium-rich borides were 1089 °C and 1260–1630 °C, respectively. Accordingly, since the formation temperature of chromium borides through the binary eutectic reaction was much higher than that of the binary nickel–boron eutectic reaction, there was a greater possibility of forming nickel-rich boride due to boron rejection. Consequently, according to the bonded sample at 1010 °C/10 min, the boron-rich residual melt was transformed into L→α(Ni) + Ni_3_B + L(rem). The formation of nickel-rich boride enriched the remaining melt with chromium and silicon. The low solubility of chromium in nickel boride (10.11 at. %) and solid solution γ (18 at. %) enriched the remaining melted materials of chromium [39]. Therefore, the conditions were present to perform a ternary eutectic reaction: L→α(Ni) + Ni_3_B + CrB + L(rem). The formation of the boride phases resulted from the rejection of silicon into the remaining melt because of the insolubility of silicon in nickel- and chromium-rich borides. The less-than-unity distribution coefficient of silicon in nickel, along with a very small amount of boron in the remaining melt, caused the interlayer to be solidified due to the ternary eutectic reaction: L→α(Ni) + Ni_3_B + Ni_6_Si_2_B. The X-ray diffraction pattern from the fracture surface of the 1050 °C/10 min sample is presented in Figure 15b and it confirmed the formation of chromium-rich boride. 

Figure 13c shows the SEM micrograph of the TLP-bonded sample at 1100 °C/10 min. The extent of the isothermal solidification zones on both sides of interface considerably increased. EDX analysis of ISZ_FSX-414_ and ISZ_X-45_ (E and F, respectively) showed that the enrichment of the isothermal solidification resulted more from chromium, cobalt, tungsten, and molybdenum elements transferred from the base metals to the bonding area (Table 4). The solidification mode of the TLP-bonded sample at 1100 °C/10 min was similar to the 1050 °C/10 min one, but the intermetallic compounds were discontinuous at the bonding centerline. Figure 13d shows the SEM micrograph of the sample bonding zone for 1150 °C/10 min. The most significant phenomenon in the sample was the absence of nickel- and chromium-rich intermetallic boride compounds, which were thoroughly eliminated, and the creation of a single-phase zone. EDX analysis of the solidification zone revealed the alloying of the interlayer melt with the base metals. Each alloying element solved with lower solubility in the interlayer melt and formed a single-phase solid solution. It is very important to precisely choose the bonding temperature and holding time in reaching the single-phase isothermal solidification zone because the isothermal solidification phase is controlled by the solid-state diffusion of melting point depressant elements into the base metals. Nickel-rich proeutectic γ dendrites moved to the bonding centerline as an epitaxial growth from the base metal/molten interlayer interface in the optimal temperature and time conditions. The melt solidification was completed by diffusing the solid state of the melting point depressant elements (boron and silicon) into the base metals through nickel-rich dendrites and the conditions were close to equilibrium; thus, the absence of solute rejection at the solid/liquid interface during the solidification stopped the formation of destructive phases. If there is a concentration gradient for the melting point depressant elements between the base metal and the molten interlayer, the isothermal solidification process will continue so that the concentration of melting point depressant elements in the bonding joint is equal to their solubility in the nickel-rich solid solution matrix at the bonding temperature. Diffusion of melting point depressant elements into the base metals resulted in increasing the melting point of the interlayer. Once the boron concentration reached the solubility of boron in nickel (0.3 at. %) and the chemical compound of the interlayer was solid Ni-17.42 Cr-16Co-3.5Fe-2.31Si-2.32W- 1.26Mn-0.16Mo, it became possible to apply the software Image J to identify the melting range of the nickel-rich solid solution in the bonding joint: the solidus temperature was 1162 °C and the liquidus temperature was 1372.39 °C.

As Figure 8 indicates, increasing temperature caused microstructural changes in the bonding zone and removed the brittle intermetallic compounds, which were caused by the change in the solidification mode. At low temperatures, there was a low diffusion coefficient for the melting point depressant elements to diffuse to zones away from the base metals. These elements reacted with the strong boride-forming elements of the base metals at the adjacent surfaces and precipitated as boride. These borides acted like strong barriers and inhibited the long-range diffusion of the melting point depressant elements. Increasing the bonding temperature made it possible to obtain the conditions for breaking, decomposition, and partial dissolution of the boride deposition. Moreover, the diffusion coefficient of the melting point depressant elements was increased and, finally, the isothermal solidification rate was also increased [40].

**Figure 17:**
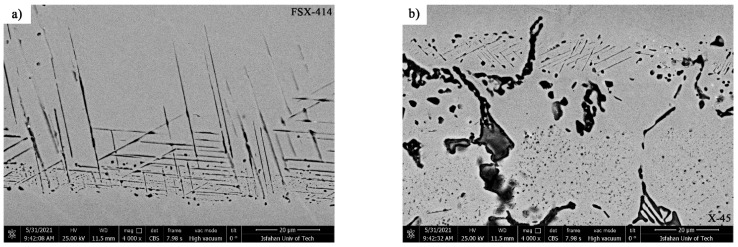
SEM micrographs of the diffusion-affected zone on the side of the (**a**) FSX-414 and (**b**) X-45 with the 1150 °C/10 min bonding condition.

**Figure 18:**
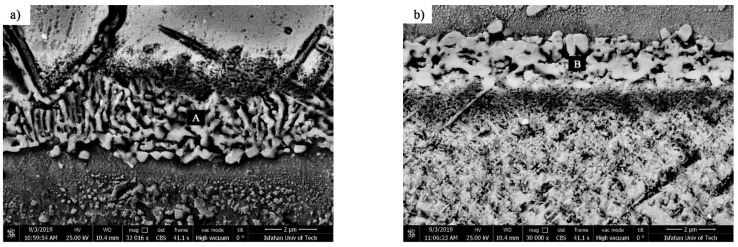
SEM micrographs of the coarse block precipitation zone on the side of the (**a**) FSX-414 and (**b**) X-45 with the 1010 °C/10 min bonding condition. Note: (A) and (B): (Ni, Cr, Co)_3_B.

**Figure 19:**
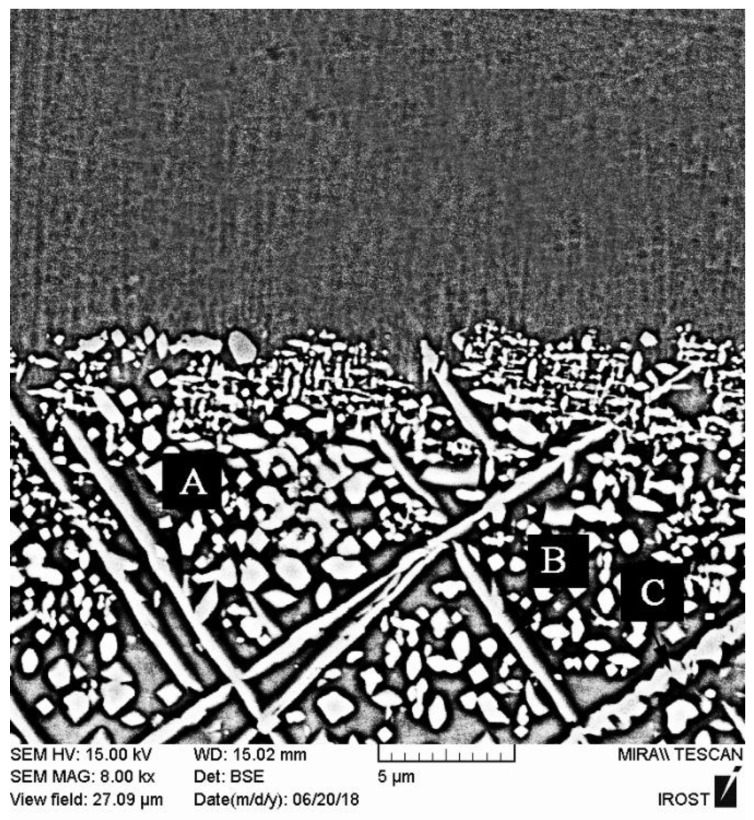
SEM micrograph of fine and needle-like precipitations on the side of the X-45 with the 1100 °C/10 min bonding condition: (A) Fine Cobalt Boride, (B) Continuous needle-like and (C) Discontinuous needle-like.

The effect of the chemical composition of the base metal directly influenced the microstructure of the diffusion-affected zone. The difference of 4% in weight between the chromium superalloys X-45 and FSX-414 made the microstructural slopes different in the side diffusion-affected zones of each base metal. Figure 17 shows the SEM micrographs of the microstructures of the diffusion-affected zones of the X-45 superalloy side and the FSX-414 superalloy side in the TLP-bonded sample with the 1150 °C/10 min condition. As the figure clearly shows, the DAZ_FSX-414_ was more extensive than the DAZ_X-45_ (56.24 vs. 13.05 μm), which was possibly due to the higher diffusion coefficient of boron in X-45 than in FSX-414 because of the increasing chromium concentration. These precipitations may have possibly precipitated and been solved in the matrix. This area had a considerably high phase density; however, close examination reveals that the diffusion-affected zone also had a microstructural slope and could be classified into three sub-zones:

(1) Coarse block precipitation zone: These precipitations were provided in the interface of the isothermally solidified zone with the base metals with a coarse block morphology. These precipitations had a more complex nature compared to other zones in the diffusion-affected zone because they were formed adjacent to the isothermally solidified zone and made it difficult to investigate. The point element analysis of these precipitations revealed that they were rich in nickel, chromium, and cobalt. Also, they carried small amounts of heavy elements, such as tungsten and molybdenum, which, due to the diffusion of boron into the base metals, makes it possible to claim that they were nickel-chromium-cobalt-rich borides. This coarse block structure had flat edges in some zones of the interface and sharp edges in other zones, which indicate their dissolution in the melt of the interlayer. Figure 18a,b show that the diffusion-affected zone was formed before the liquidus temperature of interlayer, which indicates the diffusion of the solid state during heating to the bonding temperature. These images show that the zone of coarse block precipitations was formed continuously and completely densely and allocated a significant part of the diffusion-affected zone to itself. This continuous and dense zone prevented the diffusion of boron and silicon to more inaccessible zones and stopped the spread of other zones. Increasing the bonding temperature at a constant time reduced the adherence of the coarse blocky precipitations in the zone so that it became discontinuous in the sample at 1150 °C/10 min (Figure 17), which indicated their dissolution in the melt of the interlayer. Ruiz-Vargas et al. [41] examined borides in the interface at temperatures lower and higher than the liquidus temperature of the amorphous BNi-2 interlayer. They stated that there was a low amount of boron available at low temperatures to diffuse the base metals and more inaccessible zones through the melt of the interlayer; hence, the first-generation interface borides were formed, which were caused by the accumulation of boron in the base metal/interlayer of the interface. Increasing the temperature completely melts the brazing alloy and more boron becomes available. It was necessary for the first-generation boride to be solved in order to establish the balance of the chemical composition at the molten interlayer and the base metal interfaces. The authors determined the interface borides as Ni_3_B. The borides identified in this study contained nickel, chromium, and cobalt (Table 7), and thus it is possible to claim that the interface borides were of the type (Ni, Cr, Co)_3_B.

(2) Fine and needle-like mixed-precipitation zone: This zone of precipitation was directly located inside the base metals and adjacent to the surfaces of the joint, and it involved a mixture of fine precipitations among the needle-like ones with different lengths. Figure 19 presents the SEM micrograph of the fine and needle-like mixed-precipitation zone in a sample bonded with the 1100 °C/10 min condition. Fine precipitations were scattered between the blades of the needle-like ones. EDX analysis of the fine particle showed that they were rich in cobalt, chromium, tungsten, and molybdenum (A in Table 8). Additionally, needle-like precipitations were formed with two, continuous and discontinuous, morphologies and their EDX analysis revealed that they had the same chemical compositions as the fine particles (B and C in Table 8). Consequently, these borides were rich in cobalt–chromium–tungsten–molybdenum. There were many sites inclined to concentration gradients for boron diffusion, including grain boundaries, interdendritic sites, crystalline defects (dislocations and stacking faults), and bulk material [42]. After passing the boron element from the diffusion barrier of the coarse borides of the interface, it was absorbed by the stress field of the crystalline defects in order to reduce the free energy of the system. If boron is adsorbed by the stress fields of the dislocations, it will be partitioned in these linear defects, deform the crystal lattice of the base material, and ultimately, cause the precipitation of fine borides. If boron is adsorbed by the stress fields of stacking fault defects, it will precipitate as continuous needle-like borides with a specific crystallographic direction relative to the interface of the bonding zone/metal base. Wang et al. [10] examined the formation of borides in a cobalt-based single-crystal superalloy reinforced with the γ′phase during the bonding of the transient liquid phase. They stated that, in the transient liquid phase bonding, boron diffused from the interlayer melt to the alloy matrix and was absorbed by the stacking fault defects. When the stacking fault defects were completely filled with boron, it reacted with the alloying elements cobalt, chromium, tungsten, and molybdenum to reduce the system’s free energy. Therefore, needle-like borides were formed. Unbalanced boron segregation in grain boundaries leads to the formation of discontinuous needle-like borides with the same chemical composition as continuous needle-like borides. Figure 8 shows that increasing the bonding temperature increased the area of the fine and needle-like mixed zone, which was due to the division of the interface boride barrier and the possibility of boron diffusion into more inaccessible zones.

(3) Needle-like precipitation zone: The large needle-like precipitations comprise the last zone where the boron element diffused and formed secondary phase precipitations. The formation of these precipitations showed that the boron element had diffused from the molten zone of the interlayer to zones farther from the interface through the defects of the crystal lattice and formed needle-like precipitations by reacting with strong boride-forming elements. Some of these precipitations started continuously from the fine and needle-like mixed zone and became discontinuous by progressing to the base metal at the end, which indicated the absence of sufficient boron.

Lerner et al. [43] performed X-ray diffraction and electrical resistance measurement and noted that the maximum solubility of boron in cobalt at 1000 °C is about 0.2 at. %, which is distinctly reduced when the temperature is increased such that the solubility of boron in cobalt reaches 0.16 at. % at 1095 °C. According to the cobalt–boron binary phase diagram, there is a possibility of forming borides of the cobalt system including CoB, Co_2_B, and Co_3_B. Bakhtiari at al. [17] applied an X-ray spectroscopy method based on the wavelength to quantify the boron element in the borides of the diffusion-affected zone. They recognized the amount of boron detected in the diffusion-affected zone as about 1 wt.%, which, referring to the cobalt–boron binary phase diagram, enables to state that the formation of Co_2_B and Co_3_B borides is more likely in this situation than the formation of complex (Co, Cr, W, Mo)_2_B and (Co, Cr, W, Mo)_3_B borides is in the presence of tungsten and molybdenum. The presence of these secondary phase compounds in the adjacent surfaces of the joint influenced the mechanical properties of the joint. Bakhtiari et al. [44] stated with regard to the bonding of the cobalt-based FSX-414 superalloy with a BNi-9 interlayer at 1150 °C that the fracture happened through nucleation and propagation of the crack in the diffusion-affected zone. Also, depletion of chromium in adjacent zones through the consumption of secondary phase precipitations reduces corrosion resistance, particularly at high temperatures. Consuming alloying elements of base metals in area adjacent to the bonding zone reduces the alloying elements, leading to reinforcement with a solid solution and carbide deposition (such as tungsten, molybdenum, and chromium) [44,45,46,47,48,49,50,51,52,53,54,55,56,57,58]. Consequently, it is essential to apply post-bond heat treatment to remove secondary phase compounds in the diffusion-affected zones.

## 4. Conclusions

An amorphous alloy with the analyzed composition Ni_78.92_Si_7.54_B_13.07_Fe_0.45_ was heated at different temperatures above glass transformation until melting. The DSC results showed that the crystallization of amorphous BNi-3 alloys proceeded in three stages: the first stage of crystallization (463–508 °C) was associated with the formation of solid-solution crystals based on nickel (α-Ni). The second stage (508–445.3 °C) was associated with the formation of a metastable τ-phase with an M_23_B_6_-type structure. The final stage (554.3–620 °C) involved the formation of α′-Ni + Ni3B. Cobalt-based X-45 and FSX-414 superalloys with a BNi-3 interlayer were bonded at a constant holding time of 10 min with bonding temperatures of 1010, 1050, 1100, and 1150 °C using a vacuum diffusion brazing process. Bonding at 1010 °C for 10 min caused thick centerline eutectic-like compounds at the joint, such that the bonding centerline included γ-solid solution, Ni_3_B, Ni_6_Si_2_B, and Ni_3_Si. Increasing the bonding temperature increased the dissolution of base metals and changed the solidification mode. Therefore, the athermal solidification zone of the TLP-bonded sample at 1050 °C/10 min involved γ-solid solution, Ni_3_B, CrB, Ni_6_Si_2_B, and Ni_3_Si. Finally, isothermal solidification was completed within 10 min at 1150 °C. The diffusion-affected zones on both sides had three distinct zones: a coarse block precipitation zone, a fine and needle-like mixed-precipitation zone, and a needle-like precipitation zone. Based on the EDX analysis, the coarse block precipitations were (Ni, Co, Cr)_3_B boride at the interface close to the bonding zone. The fine and needle-like (continuous and discontinuous) precipitations had the same chemical composition, so these precipitations were cobalt–chromium–tungsten–molybdenum-rich borides.

## Figures and Tables

**Table 1:** Chemical compositions of cobalt-based superalloys X-45 and FSX-414 (wt.%).

Materials	Co	Cr	Ni	W	Fe	C	Mn	Mo	B	Si
X-45	Bal.	25.41	10.47	7.48	2.13	0.24	0.26	0.55	0.010	1.27
FSX-414	Bal.	29.73	10.55	7.24	0.39	0.21	0.56	0.03	0.010	1.05

**Table 2:** Chemical composition of nickel-based BNi-3 amorphous foil (wt.%).

Material	Ni	Si	Fe	C	B
BNi-3	Bal.	4.21	0.54	0.06	2.86

**Table 3:** The energy dispersive X-ray (EDX) analysis of the various phases indicated in Figure 6 (wt.%).

Zones	Chemical Composition					Suggested Phase
	Ni	Co	Cr	Si	Fe	
A	Bal.	2.78	0.9	6.14	1.8	α-Ni
B	Bal.	2	1.1	2.97	2.4	α′-Ni
C	Bal.	0.5	0.5	0.1	1.4	Ni_3_B
D	Bal.	1.84	1.54	7.2	1.02	Ni-Si-B compounds

**Table 4:** EDX analysis of various phases indicated in Figure 13 (wt.%).

Zones	Chemical Composition							
	Co	Ni	Cr	W	Mo	Si	Fe	Mn
A	4.6	Bal.	2.2	0.5	0.12	2.98	1.1	0.15
B	6.1	Bal.	2.8	0.68	0.1	2.71	1.3	0.17
C	7.86	Bal.	9.44	1.63	0.95	2.72	1.83	0.83
D	9.24	Bal.	10.11	1.2	1.04	3.05	1.9	0.93
E	12.2	Bal.	12.65	1.83	1.1	2.34	2.12	0.89
F	13.21	Bal.	14.12	2.32	1.03	2.94	251	0.79
G	19.65	Bal.	16.23	3.67	1.52	2.2	2.98	0.93

**Table 5:** EDX analysis of various phases indicated in Figure 14 (wt.%).

Zones	Chemical Composition								Suggested Phase
	Co	Ni	Cr	W	Mo	Si	Fe	Mn	
A	0.6	Bal.	2.21	1.03	1.1	0.1	1.98	0.21	Ni_3_B
B	1.2	Bal.	1.65	1.2	0.9	0.12	2	0.18	Ni_3_B
C	0.65	Bal.	2.92	1.7	0.18	6.11	1.47	0.29	Ni_6_Si_2_B
D	4.6	Bal.	1.2	0.76	0.3	4.21	1.9	0.15	α-Ni
E	4.48	Bal.	5.4	1.13	0.71	27	0.5	0.2	Ni_3_Si

**Table 6:** EDX analysis of various phases indicated in Figure 16 (wt.%).

Zones	Chemical Composition								Suggested Phase
	Co	Ni	Cr	W	Mo	Si	Fe	Mn	
A	5.5	Bal.	5.4	1.1	0.98	0.1	0.45	0.1	Ni_3_B
B	26.6	10.30	Bal.	3.22	1.32	0.5	1.21	0.1	CrB/Cr_2_B

**Table 7:** EDX analysis of various phases indicated in Figure 18 (wt.%).

Zones	Chemical Composition								Suggested Phase
	Co	Ni	Cr	W	Mo	Si	Fe	Mn	
A	19.29	Bal.	19.71	6.98	3	0.5	2.52	0.3	(Ni, Cr, Co)_3_B
B	33.66	Bal.	18.2	6.2	2.96	0.3	2.01	0.11	(Ni, Cr, Co)_3_B

**Table 8:** EDX analysis of various phases indicated in Figure 19 (wt.%).

Zones	Chemical Composition								Suggested Phase
	Co	Ni	Cr	W	Mo	Si	Fe	Mn	
A	Bal.	11.20	26.2	3.43	0.5	1.54	1.1	1.2	Cobalt boride
B	Bal.	10.1	27.24	3.55	0.43	1.1	0.9	0.12	Cobalt boride
C	Bal.	11.21	25.1	2.67	0.58	1.01	0.89	0.29	Cobalt boride

## Data Availability

All data provided in the present manuscript are available to whom it may concern.
